# Transcriptomic Assessment of Host Responses in Vaccinia and Venezuelan Equine Encephalitis Virus-Infected Human Dendritic Cells

**DOI:** 10.3390/biom16040544

**Published:** 2026-04-08

**Authors:** Aarti Gautam, Stacy Ann Miller, Burook Misganaw, Nicholas C. Gary, Marti Jett, Sofi Ibrahim, Rasha Hammamieh

**Affiliations:** 1 Walter Reed Army Institute of Research, 503 Robert Grant Ave., Silver Spring, MD 20910, USA; stacyann.m.miller.civ@health.mil (S.A.M.); rasha.hammamieh1.civ@health.mil (R.H.); 2 Culmen International, Alexandria, VA 22314, USA; 3 U.S. Army Research Development and Engineering Command, Edgewood Chemical Biological Center, Aberdeen Proving Ground, Aberdeen, MD 21010, USA

**Keywords:** dendritic cells, Vaccinia virus, Venezuelan equine encephalitis virus, microarrays, transcriptomics, gene expression, infection, host response, canonical pathways

## Abstract

Understanding host cell response to viral infection could lead to the identification of molecular targets that can be used for the development of diagnostics and therapeutics. In this study, we investigated human dendritic cell (DC) response to infections with Vaccinia (VAC) virus, a highly immunogenic poxvirus, and Venezuelan Equine Encephalitis (VEE) virus, a single-stranded positive-strand RNA alphavirus, using human gene expression microarrays. Comparative changes in DC mRNA expression resulting from infection by the two viruses at 1, 8, and 12 h post-infection (hpi) revealed distinct temporal dynamics. VAC infection triggered early and robust activation of pathways related to chromatin organization, DNA damage, and antigen presentation, while VEE infection exhibited delayed activation of immune signaling pathways, including interferon signaling and cytokine production. Shared pathways, such as interferon signaling and inflammasome activation, highlight universal antiviral responses and potential therapeutic targets. These findings provide a molecular framework affected by VAC and VEE that need to be validated with additional experiments, such as functional assays or in vivo studies. The specific up- or downregulation of these pathways at different time points likely dictates the overall outcome of the viral infection and could potentially lead to better understanding of the temporal regulatory dynamics of virus host response.

## 1. Introduction

Dendritic cells (DCs) are potent antigen-presenting cells that play an important role in both innate and adaptive immunity [1,2,3,4]. DCs express a variety of pattern recognition receptors (PRRs) that enable detection of pathogen-associated molecular patterns upon exposure to a pathogen [5,6]. This recognition triggers the activation of signaling pathways, leading to the production of pro-inflammatory cytokines via NF-kB and type I interferons (IFNs) via interferon regulatory factors (IRFs) [7]. DCs are also critical in stimulating T cell memory, enhancing B cell proliferation and recruiting other immune cells, thereby serving as a central hub for orchestrating immune responses [1,8,9,10,11]. Given their essential role in host defense, viruses often target DCs to evade immune detection and establish infection [12,13,14]. Vaccinia (VAC) virus and Venezuelan equine encephalitis (VEE) represent two distinct viral families with fundamentally different genome structures, replication strategies, and immune evasion mechanisms.

VAC is a member of the Poxviridae family, is a large double-stranded DNA virus known for its ability to encode a wide array of immunomodulatory proteins [15,16,17]. These include virostealth proteins that reduce cell-mediated immune responses to recognize infected cells; virotransducers that inhibit innate antiviral pathways; virokines and viroceptors that block extracellular communication signals to promote antiviral immune response [16,17,18,19]. VAC infection in DCs has been shown to inhibit DC maturation, impair antigen presentation, and trigger apoptosis, thereby subverting the development of antiviral immunity [10,15,20].

In contrast, VEE is a positive, single-stranded RNA alphavirus from the Togaviridae [21,22] family and employ a different set of strategies to evade host immunity. VEE rapidly suppresses host transcription and translation through the activity of its nonstructural proteins, such as nsP2, which induces host translation shutoff and weakens the IFN-primed antiviral state. Additionally, the VEE capsid protein interferes with nuclear import, disrupting STAT1 phosphorylation and preventing the transcription of interferon-stimulated genes (ISGs). These mechanisms allow VEE to evade early immune responses and facilitate viral replication and dissemination [23,24,25,26,27,28,29].

Despite their distinct strategies, both VAC and VEE target DCs as a primary site of infection, exploiting their central role in immune activation to establish systemic infection [1,6,30,31,32,33,34,35,36,37]. Understanding how these viruses modulate DC function is critical for elucidating the molecular mechanisms of immune evasion and identifying potential therapeutic targets. The primary goal of this study was to identify host biomarkers that could inform early diagnostic or therapeutic strategies for viral infections. By leveraging transcriptomic profiling of dendritic cells (DCs) infected with Vaccinia virus (VAC) and Venezuelan equine encephalitis virus (VEE), we identified distinct and shared molecular pathways that are modulated during infection. These findings highlight several candidate biomarkers, such as interferon-stimulated genes (e.g., IFITM3, MX1, STAT1) and inflammatory mediators (e.g., CXCL9, CCL5, TNFAIP6), which were consistently altered across time points and infection types. Moreover, the temporal dynamics of host responses to VAC and VEE—where VAC induces a robust and immediate transcriptional response, while VEE exhibits a delayed and blunted response—highlight the importance of early host–pathogen interactions in determining infection outcomes.

In this study, we perform a comparative transcriptomic analysis of human DCs infected with VAC and VEE to characterize the molecular changes associated with each virus. By identifying the pathways and genes regulated by these viruses, we aim to uncover potential biomarkers and therapeutic targets that could inform the development of diagnostics and antiviral strategies. This comparative approach not only sheds light on the unique and shared mechanisms of immune modulation by VAC and VEE but also provides a framework for studying other DNA and RNA viruses that target DCs. This study is the first to directly compare the early transcriptional responses of two distinct viral families highlighting their unique and shared immune evasion strategies.

## 2. Materials and Methods

### 2.1. Viruses, Cells and Cultures

Venezuelan equine encephalitis (VEE) TC-83 and VAC Lister viruses were obtained from American Type Culture Collection (ATCC, Manassas, VA, USA). Vero cells (ATCC) were used to produce the VEE and VAC viral seeds used in this study. Vero cells were used for VEEV virus plaque assays, and BSC-40 cells (ATCC) were used for VAC plaque assays. Vero and BSC-40 cells were maintained in Dulbecco’s Modified Eagles Media (DMEM) containing 10% fetal bovine serum (FBS) and 1% penicillin/streptomycin at 37 °C/5% CO_2_. Human DCs (Astarte Biologics, Bothell, WA, USA) were maintained in Roswell-Park Memorial Institute (RPMI) 1640 media, which contained 5% heat-inactivated FBS, 2 mM L-glutamine, and 1% penicillin/streptomycin, overnight prior to viral infection.

### 2.2. Virus Infections of Dendritic Cells

Multiple vials of monocyte-derived immature dendritic cells (DCs) from 4 different donors were purchased from the supplier at 1–2 M cells/vial (Astarte Biologics, Cat # 1010). These cells were pooled and placed in 12-well culture dishes at a density of approximately 1 × 10^6^ cells per well in 500 μL of X-Vivo 15 media (Lonza, Walkersville, MD, USA). As per the manufacturer’s instructions, DCs were prepared by culturing primary monocytes in the presence of IL-4 and GM-CSF [38]. After a 20 min incubation at 37 °C/5% CO_2_, cells were infected with VAC strain Lister or VEE strain TC-83 at a multiplicity of infection (MOI) of 10, or mock-infected, in triplicates. Infected cells were harvested at 1, 8, and 12 h post-infection (hpi). Cells were routinely inspected using microscopy (Figure S1).

### 2.3. Nucleic Acids Isolations

Total RNA molecules were isolated from infected dendritic cells using AllPrep DNA/RNA/Protein kit according to the manufacturer’s instructions (Qiagen, Valencia, CA, USA). The quality and quantity of RNA were determined using NanoDrop 2000 spectrometer (Thermo Fisher, Wilmington, DE, USA) and Agilent 2100 Bioanalyzer (Agilent Technologies, Inc., Santa Clara, CA, USA). All RNA samples had an RNA integrity number (RIN) value > 9.0 (Table S1A).

### 2.4. Microarrays

All microarrays and reagents for labeling and hybridizations were purchased from Agilent Technologies (Agilent Technologies, Santa Clara, CA, USA). Expression arrays were performed on Agilent Sureprint Human Gene Expression (v2) 8 × 60 K (ID 039494) [39]. The mRNA microarray contained 50,599 biological features. All labeling, hybridization and scanning of the microarrays were performed according to the manufacturer’s protocol. For mRNA, Agilent’s two-color, two-sample procedure was carried out. The Agilent two-color low Input Quick Amp Labeling Kit, along with RNA spike-ins from Agilent’s two-color RNA spike-in kit, were used for mRNA labeling. Briefly, 200 ng of total RNA of each sample was converted to cDNA, then in vitro transcribed and labeled with Cy5-CTP. A 200 ng sample of human reference RNA (Agilent Technologies) was Cy-3-labeled and used as control. All samples were fragmented and equal amounts (300 ng) of both dye-labeled samples were co-hybridized to the Agilent-039494 Sureprint Human GE (v2) 8 × 60 K microarray using the Agilent Gene Expression Hybridization kit according to the manufacturer’s instructions for 17 h at 65 °C. After hybridization arrays were washed and scanned using an Agilent DNA Microarray Scanner (G2600D) (Agilent Technologies, Inc., Santa Clara, CA, USA), microarray data was deposited in the NCBI Gene Expression Omnibus (GEO) under the accession number GSE309062. Real-time PCR was performed to validate the microarray data. Total RNA was reverse-transcribed, and PCR reactions were performed using RT^2^ profiler PCR arrays corresponding to human antiviral response genes (Qiagen) following manufacturer’s guidelines.

### 2.5. Data Analysis

Raw signal intensities were extracted from scanned Agilent microarray images using Agilent Feature Extraction Software (v11.5.1.1). The resulting transcriptomic data analysis was conducted within the R statistical environment (version 4.0) using several packages from the Bioconductor project. For the initial processing of the mRNA data, we utilized the LIMMA (Linear Models for Microarray Data) R package. Background correction was performed based on preliminary quality assessments, and within-array normalization was performed using the LOESS algorithm (Figure S2) to account for intensity-dependent biases. To ensure high-quality data and prevent artificially inflated signals, technical replicate probes were averaged using the avereps function of LIMMA, and probes lacking associated REFSEQ or official HGNC gene symbols were filtered out prior to downstream analysis.

To evaluate the global structure of the gene expression dataset and identify primary sources of variation, we performed principal component analysis (PCA) on the scaled and centered expression matrix. We employed Prince plot analysis (via the swamp package) to quantify the association between the first ten principal components and experimental metadata. This allowed us to confirm that the interaction between infection condition and time point was the dominant driver of variance, while also verifying the absence of significant microarray chip batch effects.

Differential gene expression (DGE) was performed using a linear model with a design matrix representing the 1, 8, and 12 h time points for each infection condition (VAC, VEE, and Control). Contrast matrices were constructed to perform comparisons of (1) infected cells versus their time-matched controls, and (2) VAC-infected versus VEE-infected cells at each interval. To improve the stability of variance estimates, especially for genes with low expression, we applied the empirical Bayes (eBayes) method. Statistical significance was defined using an adjusted *p*-value < 0.05 with the Benjamini–Hochberg False Discovery Rate (FDR) procedure applied to correct for multiple testing as well as an absolute log2 fold-change (|logFC|) > 1.

Functional enrichment and pathway analyses were conducted using Ingenuity Pathway Analysis (IPA, Qiagen). Significantly differentially expressed genes (DEGs) were mapped to the Ingenuity Knowledge Base to identify enriched canonical pathways and generate biological networks. Pathway enrichment was assessed using the right-tailed Fisher’s exact test, with Benjamini–Hochberg correction applied to the resulting *p*-values to determine the statistical significance with multiple testing correction. The output files were exported to GraphPad Prism v10 for generating figures.

## 3. Results

### 3.1. Principal Component Analysis (PCA)

**Figure 1:**
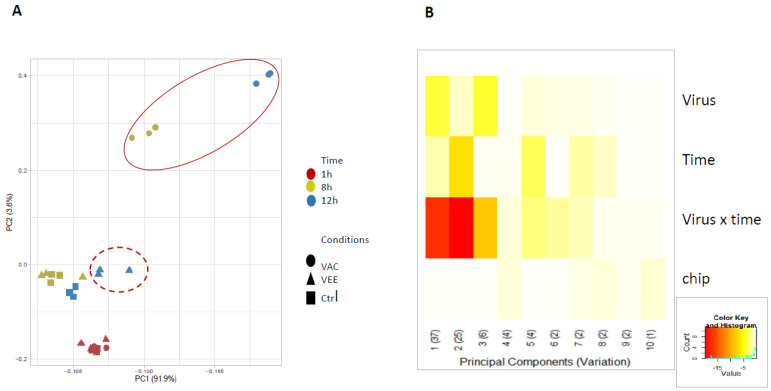
Principal component analysis (PCA) of global transcriptomic profiles of VAC- and VEE-infected human dendritic cells. (**A**) PCA scatter biplot of global mRNA expression across all time points and experimental groups. Primary human monocytic dendritic cells (DCs) were pooled from 4 donors and infected in triplicate (n = 3 per condition/time point) with VAC, VEE, or Ctrl (mock-infected). VAC-infected samples (solid circles) demonstrate a rapid and robust transcriptomic divergence from controls starting at 8 h post-infection (hpi), as highlighted by the solid red ellipse. In contrast, VEE-infected samples (solid triangles) show a delayed response, only separating significantly from time-matched controls at 12 hpi (dotted red ellipse). (**B**) Prince plot showing the association between metadata variables and the first 10 principal components. Color intensity represents the significance of the association (*p*-value, log10 scale), indicating that the interaction of infection condition and time is the primary driver of variance in PC1 and PC2, with no significant chip batch effects observed.

PCA was performed on the mRNA expression (VAC- and VEE-infected cells at different time points) dataset to visualize the global expression patterns across samples and identify major sources of variation. The resulting PCA plot (Figure 1A) reveals a clear clustering pattern primarily driven by interaction of the infection type/condition (VAC, VEE, Ctrl) and the time point at which the data was collected. Data points from the 1 h, 8 h, and 12 h time points form distinct clusters, suggesting that time is a strong factor influencing the observed variance. At 1 h, samples are largely intermixed across experimental conditions, with some separation seen in VAC-infected cells in the first PC. By 8 h, we see some separation of VEE from controls while VAC gene expression has very clearly diverged. This trend continues at 12 h, with VEE now shifted (down and to the right) in both PC1 and 2 from the controls and VAC further clustered away. Notably, VAC at 8 and 12 hpi seems to have larger effects as observed by clusters distinct from control and earlier time points as highlighted by the ellipse in Figure 1, This indicates a unique data profile at these time points. Both PC1 (91.9%) and PC2 (3.6%) were able to capture variation with time point separation. These patterns can also be seen in the prince plot (Figure 1B) quantifying the apparent signal for virus condition, time, virus x time, and microarray chip batch effects in the first 10 principal component dimensions. While condition (VEE, VAC, or Ctrl) has a significant signal in PC1 and time in PC2, the interaction/combination of condition across time is very highly significant in both PC1 and PC2. While the 8 microarrays on each Agilent chip show some idiosyncratic co-variation, we see no evidence for significant chip batch effects in this experiment (Figure S2).

### 3.2. Differential Expression Analysis

**Figure 2:**
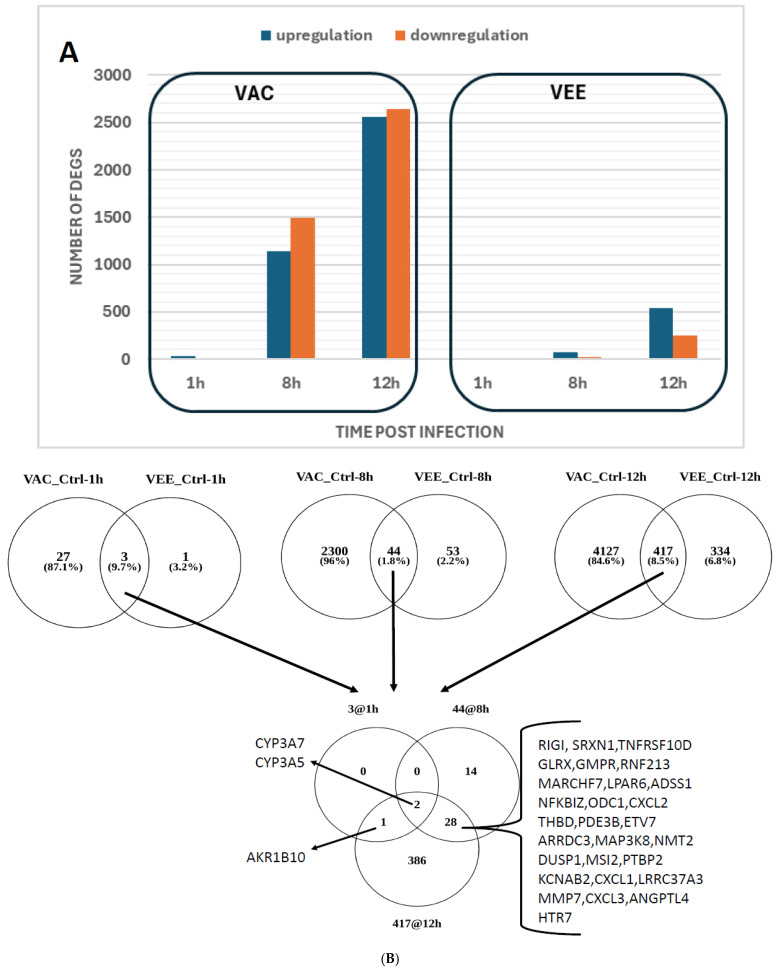
Temporal dynamics and overlapping DEG sets. (**A**) Bar plot displaying the total count of upregulated (blue) and downregulated (orange) mRNA probes at 1, 8, and 12 hpi. Significance was defined as an absolute log2 fold-change of at least 1 and a Benjamini–Hochberg-adjusted *p*-value (FDR) < 0.05. VAC infection exhibits a progressive, large-scale transcriptional response, peaking at 12 hpi with 5194 DEGs. VEE infection demonstrates a significantly more muted response, with substantial changes (796 DEGs) emerging only at 12 hpi. (**B**) Venn diagrams illustrate the overlap between VAC and VEE responses at each time point. The bottom panel identifies a “core” response of 28 genes shared between the two infections across both 8 and 12 hpi, with 2 genes (*CYP3A7* and *CYP3A5*) being consistently altered across all conditions and time points.

Even if the control samples showed minimal gene expression changes across time points as expected, we still focused on time-matched controls to look for VEE- and VAC-specific patterns of expression. Expression levels of several genes significantly changed over time in VEE and VAC and both. There was progressive change in VAC infection, and VEE had some changes mainly at the 12 h time point post-infection. The volcano plots of differential gene expression across time points in VEE and VAC infection are shown as Figure S3. The detailed list with differentially expressed genes for each of the treatment at respective time points is provided (Table S1B,C). Figure 2A shows the number of unique mRNA probes, which are significantly (corrected *p*-value < 0.05) upregulated and downregulated at each time for VEE vs. Ctrl and VAC vs. Ctrl comparisons. Minimal changes were observed in VEE infection at 1 hpi with five upregulated genes being significantly altered. By 8 hpi, the number of significantly expressed genes were 99 with 78 upregulated and 21 downregulated. The most striking gene expression was observed at 12 hpi, where the number of significantly regulated genes were 796 (542 upregulated and 254 downregulated) in the VEE-infected cells. Similarly, there was a gradual increase in gene expression changes in VAC infection at 8 hpi and 12 hpi. At 1 hpi, the number of significantly expressed genes in VAC-infected cells was 32 upregulated genes. By 8 hpi, the number of significantly expressed genes were 2630 (1138 upregulated and 1492 downregulated) and 5194 (2557 upregulated and 2637 downregulated) at 12 hpi in VAC-infected cells. The Venn diagrams comparing the overlapping genes between VEE and VAC at different time points (1-, 8-, and 12 hpi), illustrating the changes in genes between these groups over time, are shown in Figure 2B. Two genes, *CYP3A7* and *CYP3A5*, were consistently changing across time points in both VEE and VAC infections.

### 3.3. Altered Pathways in Responses to In Vitro VEEV or VACV Infection

**Figure 3:**
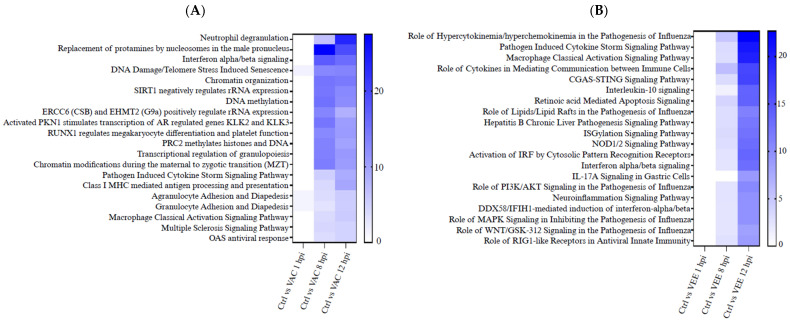
Temporal dynamics of enriched canonical pathways in VAC- and VEE-infected human monocyte-derived DCs. Heatmaps representing the top 20 canonical pathways identified via Ingenuity Pathway Analysis (IPA) for (**A**) VAC-infected and (**B**) VEE-infected monocyte-derived DCs (compared to time-matched controls). Enriched pathways are ranked by significance *p*-values. Pathways enriched in VAC at 8–12 hpi are dominated by fundamental cellular processes including chromatin organization, DNA damage response, and macromolecule synthesis. VEE-specific enrichment, which is most pronounced at 12 hpi, are related to innate immune signaling, including interferon signaling, cytokine storms, and pattern recognition receptor (PRR) activation.

Minimal transcriptomic responses were observed in VAC and VEE infections at 1 hpi. VAC infected cells had strong responses at 8 and 12 hpi and were primarily concerned with fundamental cellular processes related to DNA and chromatin organization, gene regulation, and early development. The key theme here was related to canonical pathways such as chromatin modification, transcriptional regulation in specific cell types (e.g., granulopoiesis, megakaryocytes), DNA damage response, and early developmental events (maternal to zygotic transition) (Figure 3A and Table S2). Also, this included some immune-related pathways like neutrophil degranulation and antigen presentation. For the VEE infection, the primary pathways were concerned with immune signaling pathways, particularly those involved in viral infections and inflammatory responses. Here, the key theme was cytokine signaling (interferons, interleukins), pathogen recognition (e.g., CGAS-STING, NOD1/2, RIG-I-like receptors), antiviral responses, inflammation (cytokine storm, neuroinflammation), and specific diseases (e.g., Hepatitis B). This also includes involvement of lipids and various signaling molecules (PI3K/AKT, MAPK, WNT) (Figure 3B and Table S3). A lower response was observed at 8 hpi VEEV-infected cells enriched at 12 hpi. Many significantly up- and downregulated genes included IFN-related genes (IFNB11), chemokine (CCL5 and CXCL11), and others. TNF, STAT1, and AIM2 were used for validation using alternate methods such as real-time PCR and show concordance for most of the time points (Figures S4 and S5).

### 3.4. Similarities and Differences Between VEEV and VACV Infection-Induced DEGs

**Figure 4:**
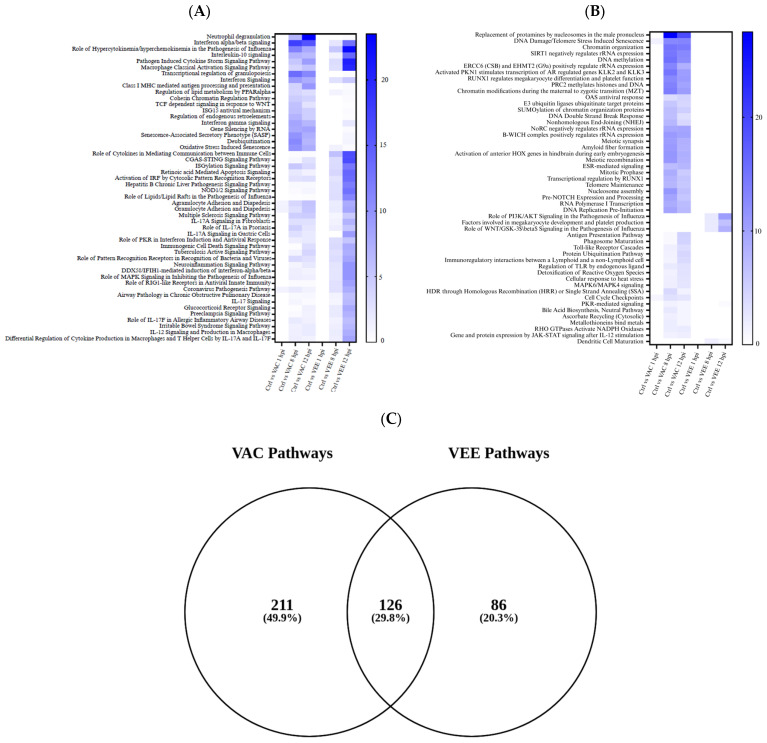
Comparisons of canonical pathways enriched in VAC and VEE infections over time. (**A**) Heatmap of top 50 canonical pathways commonly enriched across both infection types, highlighting a universal host response to viral exposures. (**B**) Heatmap of top 50 canonical pathways uniquely enriched in either VAC or VEE infection, demonstrating the divergent host responses to these distinct viruses. (**C**) Venn diagram showing that while 126 canonical pathways are shared, VAC infection modulates a significantly broader range of pathways (211 unique) compared to the 86 unique ones for VEE infection. Enrichment significance was determined at *p* < 0.001.

The results of gene enrichment analysis were compared between the VEE and VAC responses at all examined time points (Figure 4 and Table S4). The common canonical pathways were heavily focused on immune signaling, inflammation, and disease pathogenesis, while it is still retaining elements of DNA regulation and cellular processes. While VAC and VEE infections exhibit distinct transcriptional profiles, this study also highlights several shared pathways that are central to the host’s response to viral infections. These shared pathways reflect the host’s universal antiviral defense mechanisms, which are activated in response to diverse viral pathogens The major differences included activation of interferon signaling pathways, cytokine production (e.g., IL-10, IL-17A), and downstream signaling cascades mediated by PI3K/AKT, MAPK, and WNT significant only after VAC infection. Specific pathways are involved in pathogen recognition (CGAS-STING, NOD1/2, RIG-I-like receptors) and antiviral responses. VAC infection also triggered disease-relevant pathways, specifically those associated with viral infections (e.g., Hepatitis B, Influenza) and inflammatory disorders. The role of lipid rafts signaling in influenza pathogenesis seemed to be altered in VEE at higher intensity. Thus, commonality of the two infections involves different networks, with a greater focus on the adaptive immune response, the role of cytokines in disease and inflammation, and are oriented towards the consequences of infection and immune dysregulation. On the contrary, as seen among the top 50 networks in Figure 4 and Table S4, the differences between both infections (VEE and VAC) were primarily involved in fundamental cellular processes, particularly those related to chromatin organization, transcriptional regulation, and early development. Key differentiating networks included higher changes in mRNA for chromatin modification (methylation by PRC2, regulation by SIRT1, ERCC6, and EHMT2), DNA damage responses, and transcriptional control in specific hematopoietic lineages (granulopoiesis, megakaryocyte differentiation regulated by RUNX1) in VAC infection. The VAC infection is also different from VEE in early developmental events (maternal to zygotic transition) and select aspects of immune function (neutrophil degranulation, antigen presentation), DNA/RNA regulation, cellular processes, and some immune signaling. Thus, it suggests that VAC infection has a larger impact on the machinery governing gene expression and genome packaging that is absent in VEE infection until 12 hpi. In addition, processes like DNA double-strand break response and telomere maintenance highlight that mechanisms safeguarding the genome are also affected byt VAC infection.

## 4. Discussion

Our study offers a side-by-side view of very early innate programs in primary human DCs challenged with a cytoplasmic DNA virus (VAC) versus a positive-strand RNA alphavirus (VEE). Across 1, 8, and 12 hpi, VAC provoked broader and earlier transcriptional activation, whereas VEE showed delayed or blunted induction that could be due to the larger genome size, with more viral antigens recognized by the host [40,41]. These kinetics are consistent with the distinct immune-evasion strategies of these virus families.

Although VAC encodes an extensive arsenal of immune antagonists, it also exposes DCs to cytosolic DNA and other cues that engage cGAS–STING and TLR pathways. The poxviral nuclease poxin degrades the second messenger 2′,3′-cGAMP, thereby limiting STING activation and enhancing viral fitness [42]. The TIR-like protein A46 targets multiple TLR/IL-1 adaptor proteins (MyD88, MAL, TRIF and TRAM), disrupting both MyD88-dependent and TRIF-dependent pathways [43,44,45,46]. The dsRNA-binding protein E3L blunts nucleic-acid-triggered IFN-β induction [47]. Functionally, VAC can inhibit DC maturation and impair antigen presentation [20]. Our observation of early induction of interferon-stimulated gene (ISG) modules, together with partial dampening, is consistent with DC sensing of viral DNA coupled to concurrent antagonism of key signaling nodes.

In contrast, VEEV deploys mechanisms that rapidly limit host gene expression and IFN signal transduction. The capsid protein can translocate to the nucleus and block RNA polymerase II-dependent transcription; mutations that disrupt this activity attenuate virulence [28]. The lack of global expression changes at 1 hpi in VEE infection is interesting, as it correlates with the known ability of alphaviruses to inhibit cellular transcription shortly after infection [48,49]. The nonstructural protein nsP2 induces host translation shutoff and weakens the IFN-primed antiviral state [29]. Beyond these global effects, VEE disrupts STAT1 phosphorylation and nuclear translocation downstream of type I and type II IFN receptors. DCs are an early in vivo target during alphavirus spread from skin to draining lymph nodes [50], but alphaviruses also differ in their ability to infect myeloid cells [23]. Together, these mechanisms provide a plausible explanation for the comparatively muted or delayed ISG induction we detect after VEEV exposure.

The shared pathways between VAC and VEE infections highlight the host’s core immune and cellular responses to viral infections. The fact that several inflammatory and interferon response genes were activated in both VAC and VEE infections highlights the importance of these factors in early antiviral defense [33,51,52,53]. These pathways reflect a balance between the host’s attempt to control the infection and the viruses’ strategies to evade or exploit these responses. Understanding these shared mechanisms provides valuable insights into the molecular basis of viral pathogenesis and identifies potential therapeutic targets for combating infections caused by these and other viruses. The existing literature highlights different mechanisms to evade, impede, or alter the host’s antiviral defenses [30,37,54] by these two viruses and underscores the complexity and intricate interplay between different functions.

The observed similarities between our DC data and previously published spleen data from Koterski et al.’s study using non-human primate [24] are particularly encouraging, strengthening the validity of our findings and supporting the existence of a core set of genes, such as HLA-DQB1, IL1RN, IRF7, CCL3, TNFAIP6, OAS1, OASL, IL18, GBP2, IFITM3, MX1, MX2, STAT1, NCAM1, CKLF, CXCL9, and STX17, that consistently respond to VEE infection across species and tissues. There were 18 common genes supporting the regulation in the same direction and only two genes HLA-DQB1 and NCAM1 in the opposite direction of regulation. These leads support to idea that some of these transcripts may be useful as diagnostic biomarkers. Further, this data supports a working model in which DCs mount robust transcriptional programs to VAC via DNA-sensing and TLR cues that are only partially counteracted by viral inhibitors, whereas VEE suppresses transcription/translation and interferes with JAK–STAT signaling quickly enough to blunt canonical ISG and inflammatory gene induction. This interpretation aligns with recent syntheses of alphavirus innate-evasion strategies and with structural/biochemical evidence for poxviral antagonists of cGAS–STING and TLR adaptors [34,55].

### Limitations and Next Steps

Microarrays provide robust quantification of predefined probes but lack isoform-level resolution compared with RNA-seq. While the multi-donor-pooled DC-specific gene expression data provides a mechanistic basis for understanding early immune responses, further studies integrating in vivo models are needed to fully explain how these transcriptional changes translate into infection outcomes. The early time window (≤12 hpi) in primary DCs constrain generalizability, and future work should validate these results at the protein level (e.g., IFIT family members, CXCL10, type I IFN) and extend the time course and strain panel to link early transcriptional programs with functional DC outcomes.

## 5. Conclusions

Early DC transcriptional trajectories diverge sharply between VACV and VEEV. VACV elicits broad innate activation even as it deploys multiple suppressors of nucleic-acid sensing; VEEV quickly curtails host transcription/translation and interferes with IFN signal transduction. These mechanistic differences highlight tractable host–virus nodes—cGAS–STING and TLR adaptors for VACV; capsid/nsP2 and downstream JAK–STAT nodes for VEEV—that may inform antiviral strategies and the design of vaccine vectors.

## Data Availability

The original contributions presented in this study are included in the article and the supplemental material files. Microarray data can be accessed from the NCBI Gene Expression Omnibus (GEO) under the accession number GSE309062. Further inquiries can be directed to the corresponding author.

## Supplementary Materials

The following supporting information can be downloaded at: https://www.mdpi.com/article/10.3390/biom16040544/s1, Figure S1: Representative phase-contrast microscopy images of primary human monocyte-derived dendritic cells (DCs) at 1, 8, and 12 h post-infection (hpi). Images compare mock-infected (Control), Vaccinia virus (VAC)-infected, and Venezuelan Equine Encephalitis virus (VEE)-infected cells. Cells were routinely monitored to ensure viability and to observe any cytopathic effects or changes in cell density across the pooled donor samples used in the study. Figure S2: mRNA data quality is represented after LOESS within-chip normalization for microarray data, enabling reliable identification of differentially expressed genes. Figure S3: Volcano plots of differential gene expression. Scatterplots illustrating the relationship between log2 fold-change (x-axis) and statistical significance (log10 *p*-value, y-axis) for (a) 1 hpi, (b) 8 hpi, and (c) 12 hpi. Each point represents a single mRNA transcript. Red dots indicate significantly up- or downregulated genes. Significance was determined using an absolute log2 FC > 1 and a Benjamini–Hochberg-adjusted *p*-value < 0.05. Some specific genes, such as histone genes (e.g., H4C6) in VAC and interferons (e.g., IFNB1) in VEE, are labeled. Figure S4: Boxplots showing the M-values for select representative genes categorized by their response patterns. The first row displays genes exclusively regulated in VAC infection (e.g., H4C6, H4C15), the second row (e.g., STAT1, CD14, IRF7) highlights VEE-responsive genes (e.g., IFNB1, IFNL1), and the third row includes genes regulated in both viral infections. Figure S5: Bar plots showing quantitative PCR (qPCR) validation of expression levels (logFC) using qPCR arrays for six key antiviral and pro-inflammatory genes (AIM2, CCL5, CXCL11, IFNB1, STAT1, and TNF). The top panel shows responses in VAC-infected cells and the bottom panel in VEE-infected cells across 1, 8, and 12 hpi. qPCR fold changes were calculated using the 2^-DDC_T method. The high degree of concordance between the two platforms (technique: red = microarray, cyan = qPCR) reinforces the validity of the global transcriptomic findings. Table S1: (A) RNA quality and concentration information for each sample generated in the experiment. Sample ID: The unique identifier assigned to each sample. Tube ID: The corresponding tube identifier used for sample storage and processing. RNA conc. (ng/µL): The concentration of RNA in each sample, measured in nanograms per microliter (ng/µL), indicating the yield of RNA extraction. RIN (RNA Integrity Number): A measure of RNA quality, with values ranging from 1 (degraded RNA) to 10 (high-quality RNA). (B) Differentially expressed genes list from all comparisons in VAC groups. These tables provide detailed information about the fields used in gene expression analysis. Each row corresponds to a specific probe or gene, with the following attributes: ProbeName: The unique identifier for the specific probe on the microarray chip used to measure gene expression. SystematicName: A standardized name or identifier for the probe or gene, often based on the microarray platform or database. ensembl_gene_id: The unique identifier for the gene in the Ensembl database, linking the probe to detailed gene annotations such as genomic location and function. entrezgene_id: The unique identifier for the gene in the NCBI Entrez Gene database, providing cross-references to gene function, pathways, and associated diseases. hgnc_symbol: The official gene symbol assigned by the HUGO Gene Nomenclature Committee (HGNC), representing the standard, human-readable name for the gene. agilent_sureprint_g3_ge_8 × 60 k: The specific microarray platform used for the analysis, indicating the technology and design of the microarray. logFC (Log Fold Change): The logarithm (base 2) of the fold change in gene expression between two conditions (e.g., infected vs. control). Positive values indicate upregulation, while negative values indicate downregulation. AveExpr (Average Expression): The average expression level of the gene across all samples in the dataset, providing a baseline measure of expression strength. t (t-statistic): The t-statistic from a statistical test used to assess whether the gene is differentially expressed. Higher absolute values indicate stronger evidence for differential expression. P.Value: The *p*-value from the statistical test, representing the probability of observing the data if there were no true difference in expression. Smaller values suggest stronger evidence for differential expression. adj.P.Val (Adjusted *p*-Value): The *p*-value adjusted for multiple comparisons using methods like the Benjamini–Hochberg procedure to control the false discovery rate (FDR). Adjusted *p*-values reduce the risk of false positives. B (Log-Odds Score): The log-odds score of a gene being differentially expressed, calculated using Bayesian methods. Higher values indicate greater confidence in differential expression. Group: The experimental group or condition associated with the sample (e.g., “Control,” “VAC-infected,” or “VEE-infected”), indicating the context in which the gene expression data was measured. (C) Differentially expressed genes list from all comparisons in VEE groups. Description same as Table S1B. Table S2: Canonical Pathways generated using DEGs when infected cells are compared to control samples at respective time points for VAC infection. The table represents log BH *p*-value from over-represented pathways from the gene list where and the log BH *p*-value adjusts for multiple testing to control the false discovery rate. Table S3: Canonical Pathways generated using DEGs when infected cells are compared to control samples at respective time points for VEE infection. The table represents log BH *p*-value from over-represented pathways from the gene list where and the log BH *p*-value adjusts for multiple testing to control the false discovery rate. Table S4: List of common and unique canonical pathways VAC and VEE infection identified from Tables S2 and S3. The table represents log BH *p*-value from over-represented pathways from the gene list where and the log BH *p*-value adjusts for multiple testing to control the false discovery rate. The color on the table is conditional formatting as a default setting in excel file.
