# Supplementary material for: Transcriptomic Assessment of Host Responses in Vaccinia and Venezuelan Equine Encephalitis Virus-Infected Human Dendritic Cells
Source: Biomolecules. 2026 Apr 8;16(4):544. doi: 10.3390/biom16040544 (PMC13113310; doi:10.3390/biom16040544)

Figure S1. Representative phase-contrast microscopy images of primary human monocyte-derived dendritic cells (DCs) at 1, 8, and 12 hours post-infection (hpi). Images compare mock-infected (Control), Vaccinia virus (VAC)-infected, and Venezuelan Equine Encephalitis virus (VEE)-infected cells. Cells were routinely monitored to ensure viability and to observe any cytopathic effects or changes in cell density across the pooled donor samples used in the study.

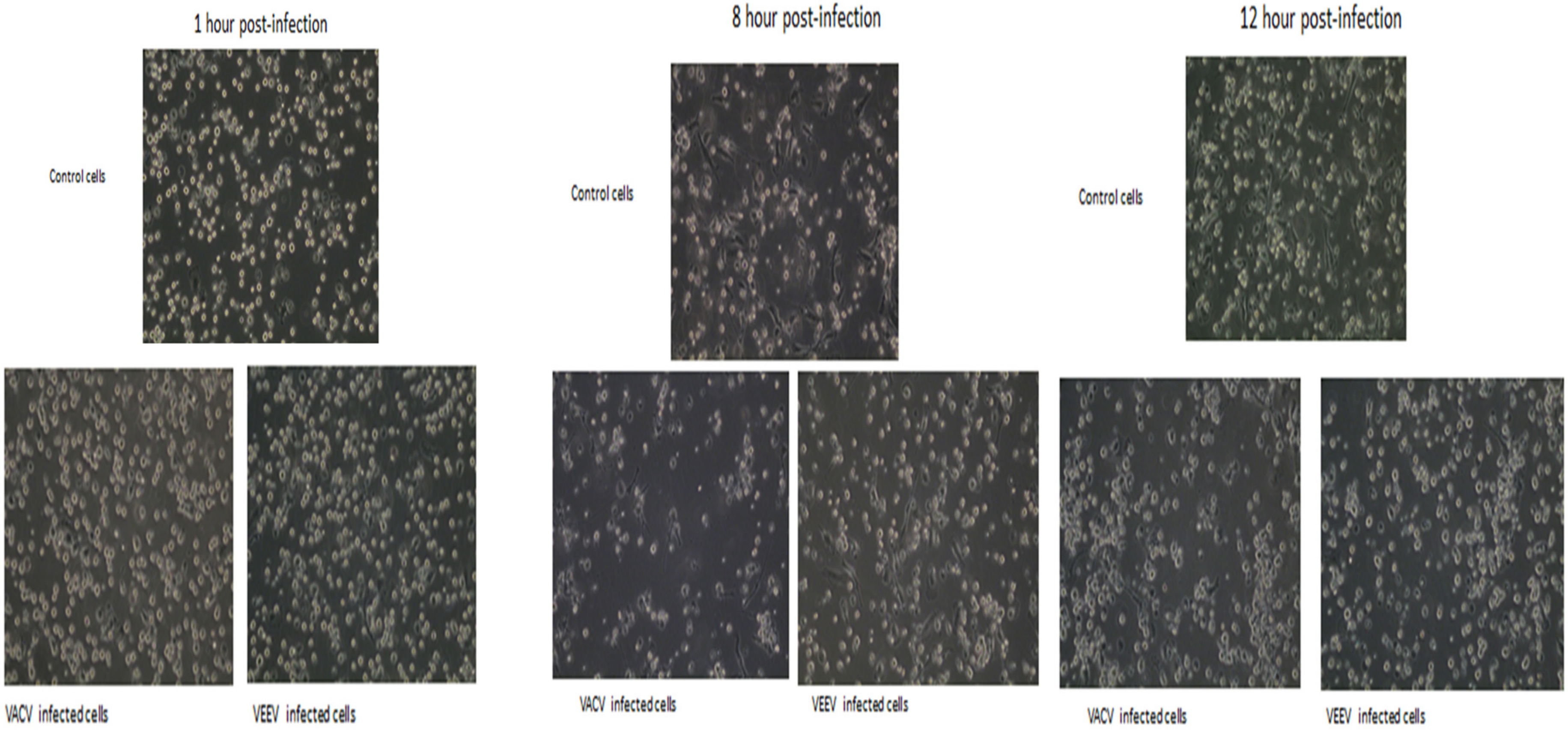

Figure S2. mRNA data quality is represented after LOESS within-Chip Normalization for microarray data enabling reliable identification of differentially expressed genes.

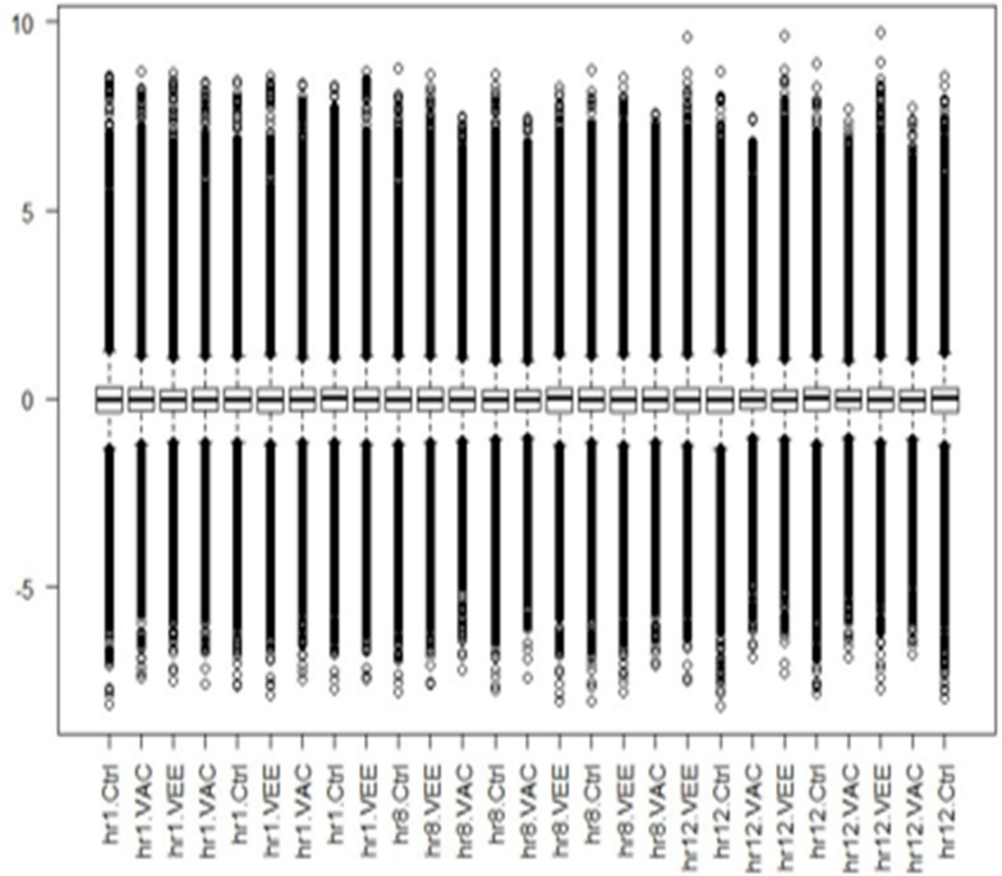

Figure S3. Volcano Plots of Differential Gene Expression. Scatterplots illustrating the relationship between log2 fold-change (x-axis) and statistical significance (log10 p-value, y-axis) for (a) 1 hpi, (b) 8 hpi, and (c) 12 hpi. Each point represents a single mRNA transcript. Red dots indicate significantly up- or down-regulated genes. Significance was determined using an absolute log2 FC > 1 and a Benjamini-Hochberg adjusted p-value < 0.05. Some specific genes, such as histone genes (e.g., H4C6) in VAC and interferons (e.g., IFNB1) in VEE, are labeled.

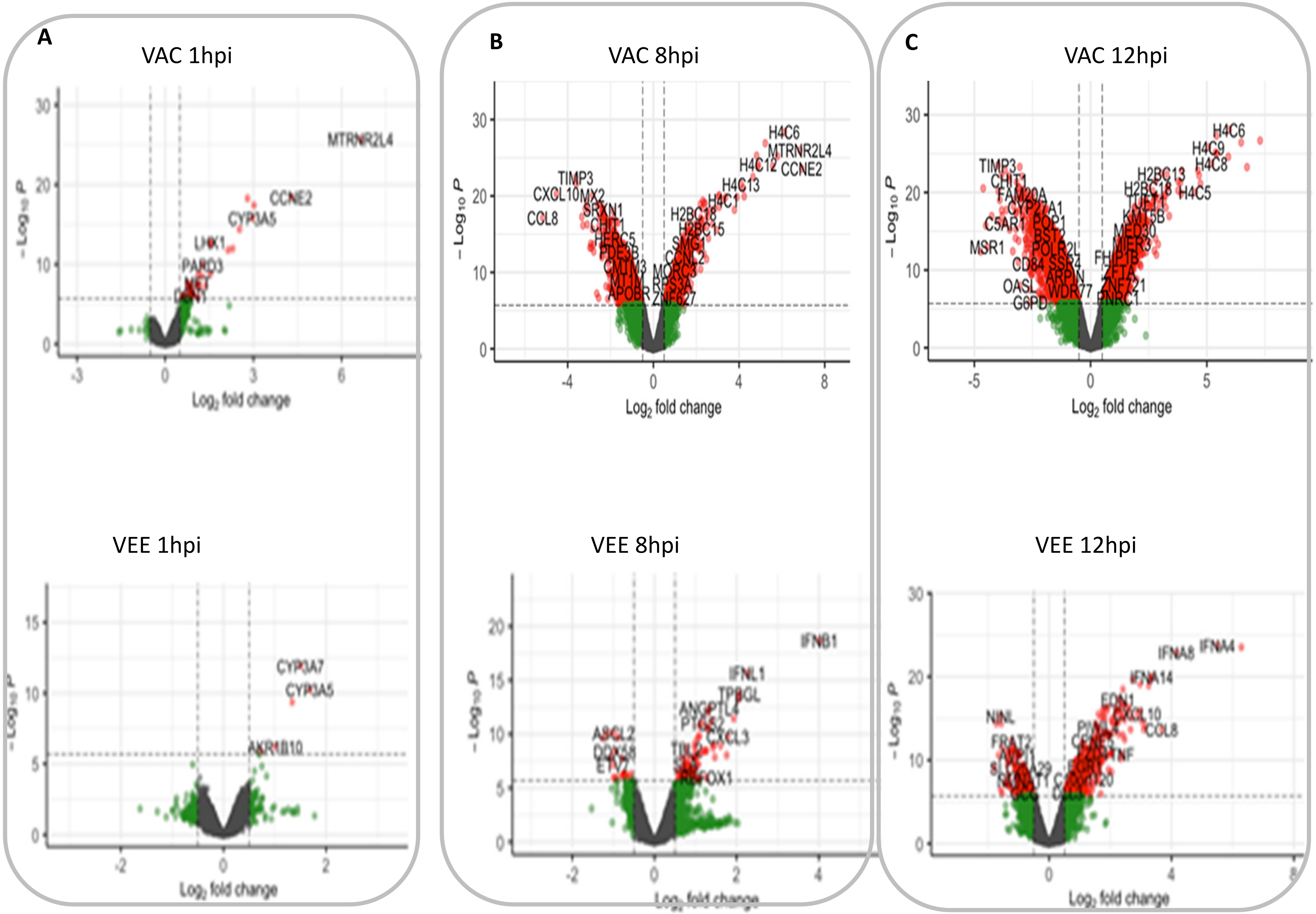

Figure S4. Boxplots showing the M-values for select representative genes categorized by their response patterns. The first row displays genes exclusively regulated in VAC infection (e.g., H4C6, H4C15), the second row (e.g., STAT1, CD14, IRF7) highlights VEE-responsive genes (e.g., IFNB1, IFNL1), and the third row includes genes regulated in both viral infections.

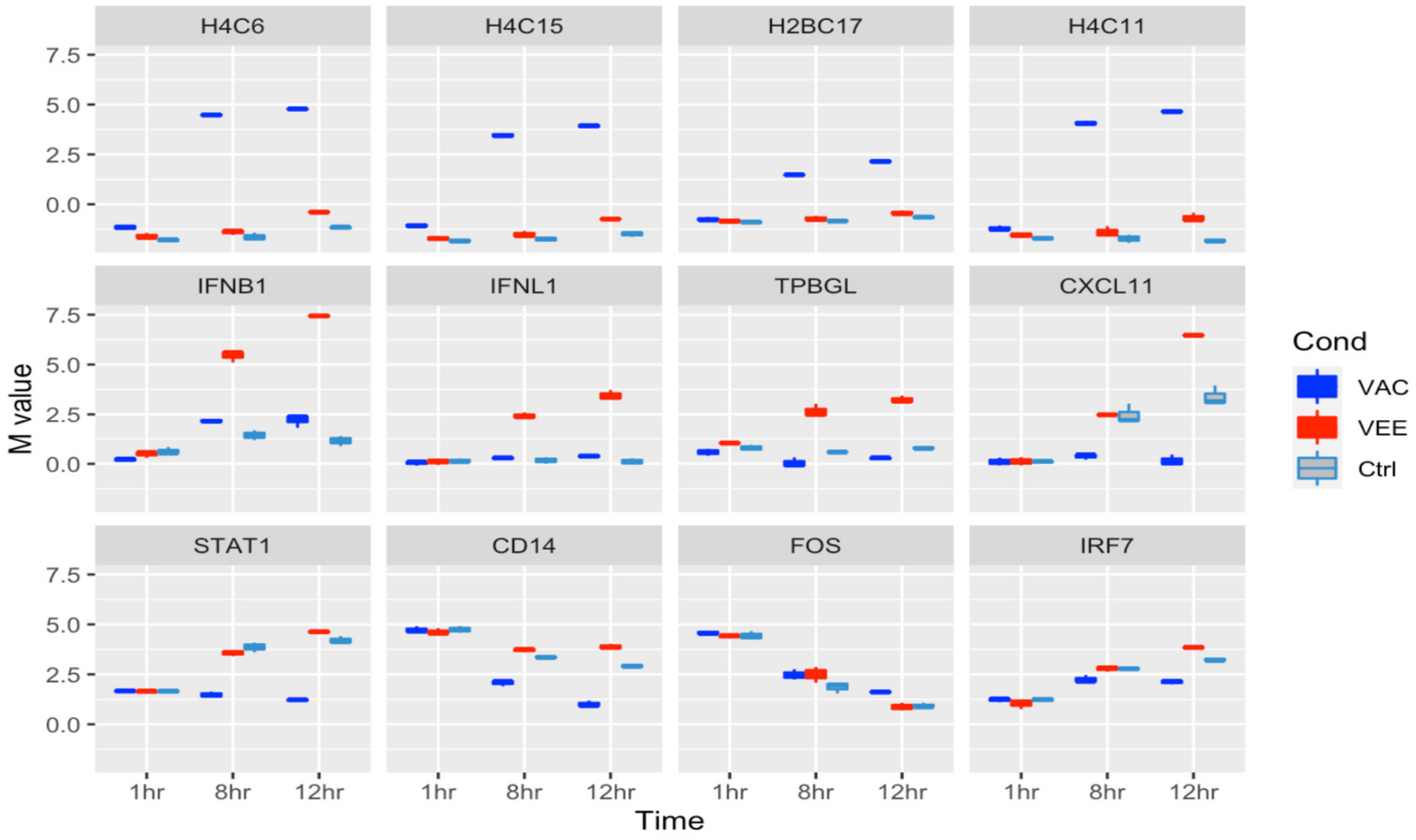

Figure S5. Bar plots showing quantitative PCR (qPCR) validation of expression levels (logFC) using qPCR arrays for six key antiviral and pro-inflammatory genes (AIM2, CCL5, CXCL11, IFNB1, STAT1, and TNF). The top panel shows responses in VAC-infected cells and the bottom panel in VEE-infected cells across 1, 8, and 12 hpi . qPCR fold changes were calculated using the 2^-DDC_T method. The high degree of concordance between the two platforms (technique: red = microarray, cyan = qPCR) reinforces the validity of the global transcriptomic findings.

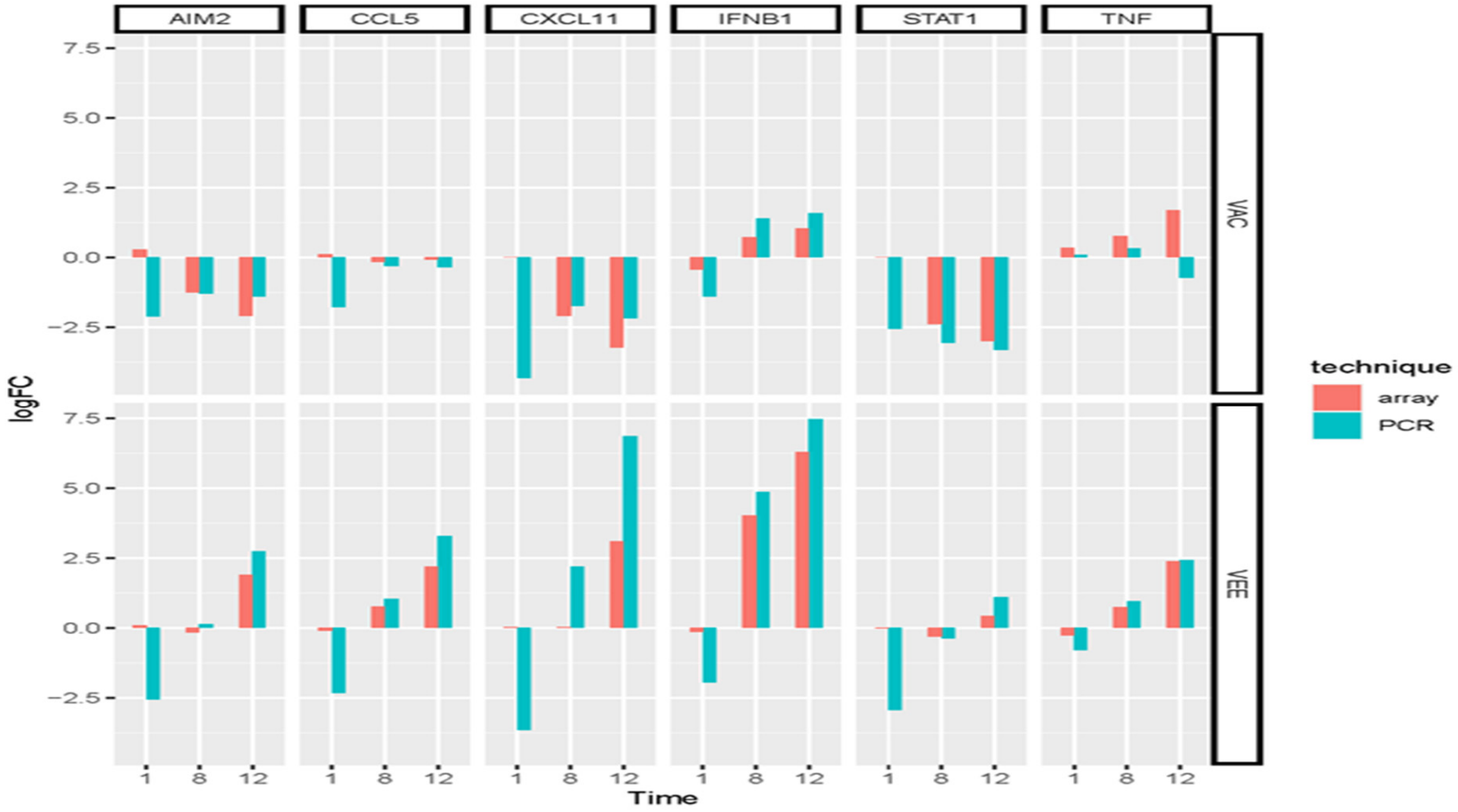

Supplement: Supplementary file 1 [file biomolecules-16-00544-s001.zip › Manuscript supplemntary figures 03042026.pdf]
